# Tetra­kis[μ-4-(diethyl­amino)benzoato-κ^2^ *O*:*O*′]bis­[(*N*,*N*-diethyl­nicotinamide-κ*N* ^1^)zinc(II)]

**DOI:** 10.1107/S1600536809027986

**Published:** 2009-07-18

**Authors:** Tuncer Hökelek, Filiz Yılmaz, Barış Tercan, Özgür Aybirdi, Hacali Necefoğlu

**Affiliations:** a Department of Physics, Hacettepe University, 06800 Beytepe, Ankara, Turkey; b Department of Chemistry, Faculty of Science, Anadolu University, 26470 Yenibağlar, Eskişehir, Turkey; c Department of Physics, Karabük University, 78050 Karabük, Turkey; d Department of Chemistry, Kafkas University, 63100 Kars, Turkey

## Abstract

In the centrosymmetric binuclear title complex, [Zn_2_(C_11_H_14_NO_2_)_4_(C_10_H_14_N_2_O)_2_], the two Zn^II^ ions [Zn⋯Zn = 2.8874 (3) Å] are bridged by four 4-(diethyl­amino)benzoate (DEAB) ligands. The four nearest O atoms around each Zn^II^ ion form a distorted square-planar arrangement, the distorted square-pyramidal coordination being completed by the pyridine N atom of an *N*,*N*-diethyl­nicotinamide (DENA) ligand at a distance of 2.0484 (12) Å. The dihedral angle between the benzene ring and the carboxyl­ate group is 4.89 (6)° in one of the independent DEAB ligands and 7.13 (7)° in the other. The benzene rings of the two independent DEAB ligands are oriented at a dihedral angle of 86.58 (5)°. The pyridine ring is oriented at dihedral angles of 31.17 (4) and 58.38 (4)° with respect to the two benzene rings. In the crystal, weak inter­molecular C—H⋯O inter­actions link the mol­ecules into a three-dimensional network. Two weak C—H⋯π inter­actions are also present. The two ethyl groups of one of the DEAB ligands are disordered over two orientations, with occupancy ratios of 0.798 (5):0.202 (5) and 0.890 (5):0.110 (5).

## Related literature

For general background to transition metal complexes of nicotinamide, one form of niacin, and/or the nicotinic acid derivative *N*,*N*-diethyl­nicotinamide, see: Bigoli *et al.* (1972 ▶); Krishnamachari (1974 ▶). For related structures, see: Hökelek *et al.* (1995 ▶); Speier & Fulop (1989 ▶); Usubaliev *et al.* (1980 ▶).
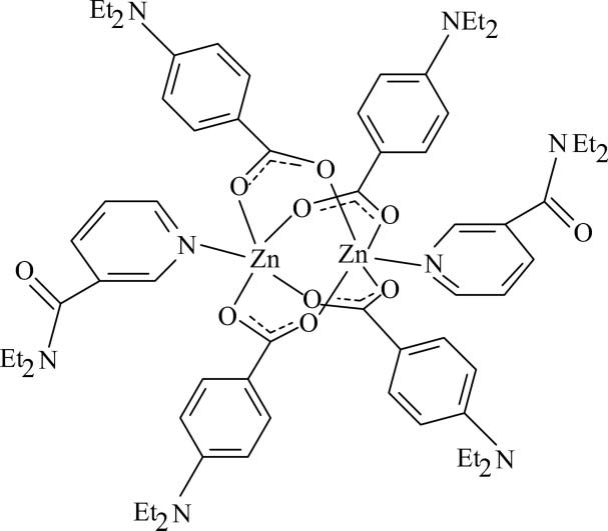


## Experimental

### 

#### Crystal data

[Zn_2_(C_11_H_14_NO_2_)_4_(C_10_H_14_N_2_O)_2_]
*M* *_r_* = 1256.13
Monoclinic, 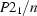

*a* = 10.3758 (2) Å
*b* = 13.4107 (2) Å
*c* = 22.4458 (3) Å
β = 93.837 (3)°
*V* = 3116.26 (9) Å^3^
*Z* = 2
Mo *K*α radiation
μ = 0.83 mm^−1^
*T* = 100 K
0.54 × 0.31 × 0.27 mm

#### Data collection

Bruker Kappa APEXII CCD area-detector diffractometer
Absorption correction: multi-scan (*SADABS*; Bruker, 2005 ▶) *T*_min_ = 0.778, *T*_max_ = 0.798
28538 measured reflections
7635 independent reflections
6141 reflections with *I* > 2σ(*I*)
*R*_int_ = 0.028

#### Refinement

*R*[*F* ^2^ > 2σ(*F* ^2^)] = 0.029
*wR*(*F* ^2^) = 0.077
*S* = 1.04
7635 reflections
417 parameters
30 restraints
H-atom parameters constrained
Δρ_max_ = 0.34 e Å^−3^
Δρ_min_ = −0.50 e Å^−3^

### 

Data collection: *APEX2* (Bruker, 2007 ▶); cell refinement: *SAINT* (Bruker, 2007 ▶); data reduction: *SAINT*; program(s) used to solve structure: *SHELXS97* (Sheldrick, 2008 ▶); program(s) used to refine structure: *SHELXL97* (Sheldrick, 2008 ▶); molecular graphics: *ORTEP-3 for Windows* (Farrugia, 1997 ▶); software used to prepare material for publication: *WinGX* (Farrugia, 1999 ▶) and *PLATON* (Spek, 2009 ▶).

## Supplementary Material

Crystal structure: contains datablocks I, global. DOI: 10.1107/S1600536809027986/ci2853sup1.cif

Structure factors: contains datablocks I. DOI: 10.1107/S1600536809027986/ci2853Isup2.hkl

Additional supplementary materials:  crystallographic information; 3D view; checkCIF report

## Figures and Tables

**Table 1:** Selected bond lengths (Å)

Zn1—O1	2.0349 (10)
Zn1—O2	2.0251 (10)
Zn1—O3	2.0465 (10)
Zn1—O4	2.0337 (10)
Zn1—N1	2.0484 (12)

Symmetry code: (i) 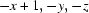.

**Table 2:** Hydrogen-bond geometry (Å, °)

*D*—H⋯*A*	*D*—H	H⋯*A*	*D*⋯*A*	*D*—H⋯*A*
C10—H10*A*⋯O5^ii^	0.99	2.49	3.390 (2)	151
C21—H21*B*⋯*Cg*1^iii^	0.99	2.94	3.879 (3)	163
C29—H29*A*⋯*Cg*1^iv^	0.99	2.87	3.637 (2)	137

Symmetry codes: (ii) 
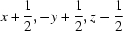
; (iii) 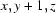; (iv) 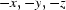. *Cg*1 is the centroid of the C2–C7 ring.

**Fig. 1.**
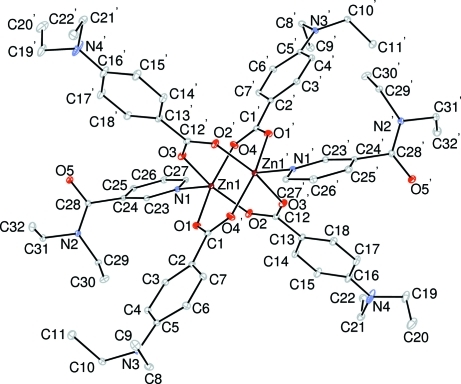
The molecular structure of the title compound with the atom-numbering scheme. Displacement ellipsoids are drawn at the 30% probability level. Only the major disorder components are shown for clarity. Primed atoms are generated by the symmetry operator:(') 1 - x, -y, -z.

## supplementary crystallographic information

### Comment

As a part of our ongoing investigation on transition metal complexes of nicotinamide (NA), one form of niacin (Krishnamachari, 1974), and/or the nicotinic acid derivative *N*,*N*-diethylnicotinamide (DENA), an important respiratory stimulant (Bigoli *et al.*, 1972), the title compound was synthesized and its crystal structure is reported herein.

The title compound is a binuclear compound, consisting of two DENA and four diethylaminobenzoate (DEAB) ligands. The structures of similar complexes of the Cu^2+^ ion, [Cu(C_6_H_5_COO)_2_(C_5_H_5_N)]_2_ (Usubaliev *et al.*, 1980); [Cu(C_6_H_5_CO_2_)_2_(py)]_2_ (Speier & Fulop, 1989) and [Cu_2_(C_6_H_5_COO)_4_(C_10_H_14_N_2_O)_2_] (Hökelek *et al.*, 1995) have also been determined. In these structures, the benzoate ion acts as a bidentate ligand.

The title dimeric complex, [Zn_2_(DEAB)_4_(DENA)_2_], has a centre of symmetry and two Zn^II^ atoms are surrounded by four DEAB groups and two DENA ligands. The DENA ligands are coordinated to Zn^II^ ions through pyridine N atoms only. The DEAB groups act as bridging ligands. The Zn···Zn' distance is 2.8874 (3) Å. The average Zn—O distance is 2.0351 (10) Å, and four O atoms of the bridging DEAB ligands around each Zn^II^ ion form a distorted square plane. The Zn^II^ ion lies 0.3229 (2) Å below the least-squares plane. The average O—Zn—O bond angle is 88.56 (4)°. A distorted square-pyramidal arrangement around each Zn^II^ ion is completed by the pyridine N atom of a DENA ligand at 2.0484 (12) Å from the Zn^II^ ion. The N1—Zn1···Zn1' angle is 171.18 (3)° and the dihedral angle between plane through Zn1, O1, O4, C1, Zn1', O1', O4', C1' and the plane through Zn1, O2, O3, C12, Zn1', O2', O3', C12' is 89.53 (5)°. The dihedral angles between the planar carboxylate groups and the adjacent benzene rings A (C2—C7) and B (C13—C18) are 4.89 (6)° and 7.13 (7)°, respectively, while that between rings A and B is A/B = 86.58 (5)°. Ring C (N1/C23—C27) is oriented with respect to rings A and B at dihedral angles A/C = 31.17 (4) and B/C = 58.38 (4)°.

In the crystal structure, weak intermolecular C—H···O interactions (Table 1) link the molecules into a three-dimensional network, in which they may be effective in the stabilization of the structure. Two weak C—H···π interactions (Table 1) are also found.

### Experimental

The title compound was prepared by the reaction of ZnSO_4_.H_2_O (0.9 g, 5 mmol) in H_2_O (50 ml) and DENA (1.78 g, 10 mmol) in H_2_O (50 ml) with sodium *p*-diethylaminobenzoate (2.16 g, 10 mmol) in H_2_O (100 ml). The mixture was filtered and set aside to crystallize at ambient temperature for one week, giving colorless single crystals.

### Refinement

The two ethyl groups attached at N4 are disordered over two orientations. During the refinement process, the disordered C19, H19A, H19B, C20, H20A, H20B, H20C and C19A, H19C, H19D, C20A, H20D, H20E, H20F atoms were refined with occupancies of 0.798 (5) and 0.202 (5), while C21, H21A, H21B, C22, H22A, H22B, H22C and C21A, H21C, H21D, C22A, H22D, H22E, H22F atoms were refined with occupancies of 0.890 (5) and 0.110 (5), respectively. The corresponding bond distances in the disorder components were restrained to be equal and the U^ij^ parameters of atoms C19A, C21A and C22A were restrained to an approximate isotropic behaviour. H atoms were positioned geometrically, with C—H = 0.95, 0.99 and 0.98 Å, for aromatic, methylene and methyl H atoms, respectively, and constrained to ride on their parent atoms, with *U*_iso_(H) = *xU*_eq_(C), where *x* = 1.5 for methyl H and *x* = 1.2 for all other H atoms.

### Crystal data

**Table:**

[Zn_2_(C_11_H_14_NO_2_)_4_(C_10_H_14_N_2_O)_2_]	*F*(000) = 1328
*M**_r_* = 1256.13	*D*_x_ = 1.339 Mg m^−^^3^
Monoclinic, *P*2_1_/*n*	Mo *K*α radiation, λ = 0.71073 Å
Hall symbol: -P 2yn	Cell parameters from 9966 reflections
*a* = 10.3758 (2) Å	θ = 2.4–28.2°
*b* = 13.4107 (2) Å	µ = 0.83 mm^−^^1^
*c* = 22.4458 (3) Å	*T* = 100 K
β = 93.837 (3)°	Block, colourless
*V* = 3116.26 (9) Å^3^	0.54 × 0.31 × 0.27 mm
*Z* = 2	

### Data collection

**Table:**

Bruker Kappa APEXII CCD area-detector diffractometer	7635 independent reflections
Radiation source: fine-focus sealed tube	6141 reflections with *I* > 2σ(*I*)
graphite	*R*_int_ = 0.028
φ and ω scans	θ_max_ = 28.3°, θ_min_ = 1.8°
Absorption correction: multi-scan (*SADABS*; Bruker, 2005)	*h* = −13→13
*T*_min_ = 0.778, *T*_max_ = 0.798	*k* = −17→14
28538 measured reflections	*l* = −29→29

### Refinement

**Table:**

Refinement on *F*^2^	Primary atom site location: structure-invariant direct methods
Least-squares matrix: full	Secondary atom site location: difference Fourier map
*R*[*F*^2^ > 2σ(*F*^2^)] = 0.029	Hydrogen site location: inferred from neighbouring sites
*wR*(*F*^2^) = 0.077	H-atom parameters constrained
*S* = 1.04	*w* = 1/[σ^2^(*F*_o_^2^) + (0.0361*P*)^2^ + 1.1231*P*] where *P* = (*F*_o_^2^ + 2*F*_c_^2^)/3
7635 reflections	(Δ/σ)_max_ = 0.001
417 parameters	Δρ_max_ = 0.34 e Å^−^^3^
30 restraints	Δρ_min_ = −0.50 e Å^−^^3^

### Special details

**Table:**

Geometry. All e.s.d.'s (except the e.s.d. in the dihedral angle between two l.s. planes) are estimated using the full covariance matrix. The cell e.s.d.'s are taken into account individually in the estimation of e.s.d.'s in distances, angles and torsion angles; correlations between e.s.d.'s in cell parameters are only used when they are defined by crystal symmetry. An approximate (isotropic) treatment of cell e.s.d.'s is used for estimating e.s.d.'s involving l.s. planes.
Refinement. Refinement of *F*^2^ against ALL reflections. The weighted *R*-factor *wR* and goodness of fit *S* are based on *F*^2^, conventional *R*-factors *R* are based on *F*, with *F* set to zero for negative *F*^2^. The threshold expression of *F*^2^ > σ(*F*^2^) is used only for calculating *R*-factors(gt) *etc*. and is not relevant to the choice of reflections for refinement. *R*-factors based on *F*^2^ are statistically about twice as large as those based on *F*, and *R*- factors based on ALL data will be even larger.

### Fractional atomic coordinates and isotropic or equivalent isotropic displacement parameters (Å^2^)

**Table:**

	*x*	*y*	*z*	*U*_iso_*/*U*_eq_	Occ. (<1)
Zn1	0.590282 (14)	−0.001300 (11)	0.051808 (7)	0.01207 (5)	
O1	0.71173 (9)	0.04299 (8)	−0.01063 (5)	0.0211 (2)	
O2	0.62210 (10)	−0.14522 (8)	0.02975 (5)	0.0224 (2)	
O3	0.51994 (10)	0.14132 (8)	0.04988 (5)	0.0224 (2)	
O4	0.42848 (9)	−0.04493 (9)	0.09108 (5)	0.0233 (2)	
O5	0.89856 (10)	0.27621 (8)	0.22536 (5)	0.0239 (2)	
N1	0.70525 (11)	0.01702 (9)	0.12883 (5)	0.0141 (2)	
N2	1.04716 (11)	0.21838 (9)	0.16475 (5)	0.0165 (3)	
N3	1.07197 (11)	0.22316 (9)	−0.20302 (5)	0.0168 (3)	
N4	0.69189 (18)	−0.58476 (12)	−0.07155 (8)	0.0523 (5)	
C1	0.68215 (13)	0.05780 (11)	−0.06557 (7)	0.0171 (3)	
C2	0.78619 (13)	0.09438 (10)	−0.10265 (6)	0.0153 (3)	
C3	0.90883 (13)	0.11536 (11)	−0.07684 (6)	0.0167 (3)	
H3A	0.9277	0.1008	−0.0358	0.020*	
C4	1.00401 (13)	0.15681 (11)	−0.10930 (6)	0.0163 (3)	
H4A	1.0862	0.1713	−0.0901	0.020*	
C5	0.98026 (13)	0.17780 (11)	−0.17073 (6)	0.0156 (3)	
C6	0.85852 (13)	0.14974 (11)	−0.19736 (6)	0.0189 (3)	
H6A	0.8412	0.1582	−0.2392	0.023*	
C7	0.76393 (13)	0.11015 (11)	−0.16366 (6)	0.0178 (3)	
H7A	0.6822	0.0934	−0.1826	0.021*	
C8	1.04919 (14)	0.23897 (12)	−0.26731 (6)	0.0209 (3)	
H8A	1.0051	0.1793	−0.2848	0.025*	
H8B	1.1339	0.2444	−0.2849	0.025*	
C9	0.96903 (15)	0.33067 (13)	−0.28572 (7)	0.0267 (4)	
H9A	0.9590	0.3346	−0.3294	0.040*	
H9B	1.0129	0.3908	−0.2699	0.040*	
H9C	0.8837	0.3256	−0.2697	0.040*	
C10	1.18098 (13)	0.27590 (11)	−0.17287 (6)	0.0178 (3)	
H10A	1.2477	0.2868	−0.2017	0.021*	
H10B	1.2193	0.2333	−0.1403	0.021*	
C11	1.14461 (15)	0.37612 (12)	−0.14676 (7)	0.0241 (3)	
H11A	1.2217	0.4073	−0.1272	0.036*	
H11B	1.0800	0.3659	−0.1174	0.036*	
H11C	1.1088	0.4196	−0.1788	0.036*	
C12	0.56282 (13)	−0.18488 (11)	−0.01543 (6)	0.0178 (3)	
C13	0.59362 (14)	−0.29028 (11)	−0.02924 (6)	0.0185 (3)	
C14	0.68952 (15)	−0.34159 (12)	0.00421 (7)	0.0231 (3)	
H14A	0.7339	−0.3092	0.0372	0.028*	
C15	0.72204 (16)	−0.43823 (12)	−0.00909 (7)	0.0281 (4)	
H15A	0.7886	−0.4709	0.0146	0.034*	
C16	0.65822 (19)	−0.48938 (12)	−0.05719 (8)	0.0314 (4)	
C17	0.55900 (16)	−0.43734 (12)	−0.09041 (7)	0.0281 (4)	
H17A	0.5123	−0.4697	−0.1227	0.034*	
C18	0.52914 (15)	−0.34049 (12)	−0.07659 (7)	0.0215 (3)	
H18A	0.4628	−0.3070	−0.1000	0.026*	
C19	0.6089 (3)	−0.6454 (2)	−0.11435 (12)	0.0390 (8)	0.798 (5)
H19A	0.5177	−0.6240	−0.1135	0.047*	0.798 (5)
H19B	0.6145	−0.7167	−0.1032	0.047*	0.798 (5)
C19A	0.6650 (8)	−0.6238 (6)	−0.1345 (3)	0.021 (2)	0.202 (5)
H19C	0.6501	−0.5692	−0.1638	0.025*	0.202 (5)
H19D	0.7350	−0.6677	−0.1469	0.025*	0.202 (5)
C20	0.6553 (3)	−0.6304 (2)	−0.17580 (13)	0.0488 (9)	0.798 (5)
H20A	0.6016	−0.6697	−0.2047	0.073*	0.798 (5)
H20B	0.7454	−0.6522	−0.1762	0.073*	0.798 (5)
H20C	0.6491	−0.5596	−0.1865	0.073*	0.798 (5)
C20A	0.5425 (8)	−0.6813 (7)	−0.1250 (4)	0.025 (2)	0.202 (5)
H20D	0.5104	−0.7120	−0.1628	0.038*	0.202 (5)
H20E	0.4770	−0.6358	−0.1111	0.038*	0.202 (5)
H20F	0.5609	−0.7335	−0.0951	0.038*	0.202 (5)
C21	0.8083 (2)	−0.63245 (14)	−0.04326 (9)	0.0270 (5)	0.890 (5)
H21A	0.8758	−0.5814	−0.0346	0.032*	0.890 (5)
H21B	0.8421	−0.6818	−0.0710	0.032*	0.890 (5)
C21A	0.7424 (11)	−0.6515 (9)	−0.0200 (5)	0.014 (3)	0.110 (5)
H21C	0.7204	−0.6266	0.0196	0.017*	0.110 (5)
H21D	0.7165	−0.7222	−0.0252	0.017*	0.110 (5)
C22	0.77785 (19)	−0.68377 (15)	0.01393 (10)	0.0317 (6)	0.890 (5)
H22A	0.8564	−0.7150	0.0321	0.048*	0.890 (5)
H22B	0.7119	−0.7350	0.0052	0.048*	0.890 (5)
H22C	0.7455	−0.6347	0.0416	0.048*	0.890 (5)
C22A	0.8816 (13)	−0.6323 (12)	−0.0329 (7)	0.023 (4)	0.110 (5)
H22D	0.9389	−0.6677	−0.0036	0.035*	0.110 (5)
H22E	0.8993	−0.5606	−0.0304	0.035*	0.110 (5)
H22F	0.8966	−0.6562	−0.0731	0.035*	0.110 (5)
C23	0.78856 (12)	0.09373 (11)	0.13414 (6)	0.0151 (3)	
H23A	0.7982	0.1353	0.1005	0.018*	
C24	0.86084 (12)	0.11458 (11)	0.18662 (6)	0.0146 (3)	
C25	0.84666 (13)	0.05239 (11)	0.23546 (6)	0.0176 (3)	
H25A	0.8948	0.0646	0.2722	0.021*	
C26	0.76207 (14)	−0.02724 (11)	0.23011 (7)	0.0177 (3)	
H26A	0.7523	−0.0710	0.2628	0.021*	
C27	0.69193 (13)	−0.04194 (11)	0.17624 (6)	0.0156 (3)	
H27A	0.6322	−0.0957	0.1727	0.019*	
C28	0.93872 (13)	0.20938 (11)	0.19367 (6)	0.0162 (3)	
C29	1.10610 (13)	0.13687 (11)	0.13207 (6)	0.0184 (3)	
H29A	1.0595	0.0740	0.1392	0.022*	
H29B	1.1970	0.1283	0.1476	0.022*	
C30	1.10256 (16)	0.15648 (13)	0.06565 (7)	0.0272 (4)	
H30A	1.1425	0.1005	0.0457	0.041*	
H30B	1.1502	0.2179	0.0583	0.041*	
H30C	1.0127	0.1638	0.0499	0.041*	
C31	1.11704 (14)	0.31346 (11)	0.17016 (7)	0.0218 (3)	
H31A	1.2063	0.3033	0.1584	0.026*	
H31B	1.1222	0.3347	0.2125	0.026*	
C32	1.05431 (16)	0.39602 (12)	0.13212 (8)	0.0309 (4)	
H32A	1.1052	0.4573	0.1376	0.046*	
H32B	0.9665	0.4077	0.1442	0.046*	
H32C	1.0507	0.3763	0.0900	0.046*	

### Atomic displacement parameters (Å^2^)

**Table:**

	*U*^11^	*U*^22^	*U*^33^	*U*^12^	*U*^13^	*U*^23^
Zn1	0.01304 (8)	0.01106 (9)	0.01221 (9)	0.00028 (6)	0.00165 (6)	−0.00082 (6)
O1	0.0216 (5)	0.0223 (6)	0.0204 (5)	−0.0014 (5)	0.0085 (4)	0.0013 (5)
O2	0.0304 (6)	0.0132 (5)	0.0238 (6)	0.0017 (5)	0.0019 (4)	−0.0040 (5)
O3	0.0274 (6)	0.0142 (5)	0.0257 (6)	0.0067 (4)	0.0026 (4)	0.0000 (5)
O4	0.0168 (5)	0.0281 (6)	0.0256 (6)	−0.0040 (5)	0.0057 (4)	0.0017 (5)
O5	0.0239 (5)	0.0195 (6)	0.0292 (6)	−0.0031 (5)	0.0091 (4)	−0.0091 (5)
N1	0.0142 (5)	0.0124 (6)	0.0160 (6)	0.0015 (4)	0.0030 (4)	−0.0008 (5)
N2	0.0143 (5)	0.0152 (6)	0.0199 (6)	−0.0008 (5)	0.0010 (5)	−0.0042 (5)
N3	0.0159 (5)	0.0193 (7)	0.0157 (6)	−0.0015 (5)	0.0039 (4)	0.0010 (5)
N4	0.0780 (12)	0.0235 (8)	0.0499 (10)	0.0278 (9)	−0.0370 (9)	−0.0196 (8)
C1	0.0198 (7)	0.0104 (7)	0.0219 (8)	0.0016 (6)	0.0075 (6)	−0.0015 (6)
C2	0.0169 (6)	0.0110 (7)	0.0186 (7)	0.0010 (5)	0.0052 (5)	0.0002 (6)
C3	0.0204 (7)	0.0141 (7)	0.0157 (7)	0.0011 (6)	0.0033 (5)	−0.0005 (6)
C4	0.0159 (6)	0.0158 (7)	0.0173 (7)	−0.0001 (6)	0.0014 (5)	−0.0003 (6)
C5	0.0161 (6)	0.0128 (7)	0.0184 (7)	0.0013 (6)	0.0053 (5)	0.0000 (6)
C6	0.0189 (7)	0.0219 (8)	0.0158 (7)	0.0000 (6)	0.0017 (5)	0.0021 (6)
C7	0.0149 (6)	0.0177 (7)	0.0210 (7)	−0.0007 (6)	0.0015 (5)	0.0005 (6)
C8	0.0193 (7)	0.0275 (8)	0.0165 (7)	−0.0014 (6)	0.0055 (6)	0.0027 (7)
C9	0.0261 (8)	0.0297 (9)	0.0240 (8)	−0.0027 (7)	−0.0007 (6)	0.0073 (7)
C10	0.0161 (6)	0.0188 (8)	0.0190 (7)	−0.0021 (6)	0.0039 (5)	0.0004 (6)
C11	0.0305 (8)	0.0205 (8)	0.0215 (8)	0.0008 (7)	0.0022 (6)	0.0001 (7)
C12	0.0207 (7)	0.0141 (7)	0.0195 (7)	0.0015 (6)	0.0080 (6)	0.0015 (6)
C13	0.0240 (7)	0.0127 (7)	0.0191 (7)	0.0027 (6)	0.0040 (6)	−0.0002 (6)
C14	0.0318 (8)	0.0168 (8)	0.0200 (8)	0.0033 (7)	−0.0039 (6)	−0.0032 (7)
C15	0.0370 (9)	0.0191 (8)	0.0266 (9)	0.0099 (7)	−0.0102 (7)	−0.0026 (7)
C16	0.0457 (10)	0.0163 (8)	0.0304 (9)	0.0118 (7)	−0.0102 (8)	−0.0068 (7)
C17	0.0375 (9)	0.0196 (8)	0.0256 (8)	0.0074 (7)	−0.0111 (7)	−0.0078 (7)
C18	0.0258 (7)	0.0172 (8)	0.0211 (8)	0.0057 (6)	−0.0022 (6)	0.0006 (6)
C19	0.048 (2)	0.0193 (14)	0.0468 (17)	0.0109 (14)	−0.0154 (15)	−0.0123 (13)
C19A	0.029 (4)	0.011 (3)	0.024 (4)	0.000 (3)	0.008 (3)	0.000 (3)
C20	0.0493 (15)	0.0474 (17)	0.0470 (19)	0.0233 (13)	−0.0163 (12)	−0.0227 (14)
C20A	0.022 (4)	0.019 (5)	0.035 (5)	−0.004 (4)	−0.005 (4)	0.004 (4)
C21	0.0295 (12)	0.0161 (9)	0.0355 (11)	0.0056 (8)	0.0039 (9)	−0.0024 (8)
C21A	0.021 (5)	0.011 (5)	0.010 (5)	0.005 (4)	−0.003 (4)	0.000 (4)
C22	0.0302 (10)	0.0230 (10)	0.0419 (13)	0.0001 (8)	0.0021 (9)	0.0014 (9)
C22A	0.017 (5)	0.022 (5)	0.030 (5)	0.000 (4)	0.001 (4)	−0.002 (4)
C23	0.0147 (6)	0.0136 (7)	0.0173 (7)	0.0009 (5)	0.0041 (5)	0.0000 (6)
C24	0.0130 (6)	0.0135 (7)	0.0176 (7)	0.0016 (5)	0.0029 (5)	−0.0032 (6)
C25	0.0183 (7)	0.0184 (8)	0.0157 (7)	0.0032 (6)	−0.0010 (5)	−0.0022 (6)
C26	0.0205 (7)	0.0163 (7)	0.0166 (7)	0.0030 (6)	0.0024 (6)	0.0027 (6)
C27	0.0161 (6)	0.0119 (7)	0.0192 (7)	0.0012 (6)	0.0033 (5)	−0.0001 (6)
C28	0.0152 (6)	0.0164 (7)	0.0168 (7)	0.0002 (6)	−0.0010 (5)	−0.0011 (6)
C29	0.0146 (6)	0.0174 (8)	0.0234 (8)	0.0015 (6)	0.0028 (5)	−0.0038 (6)
C30	0.0322 (8)	0.0264 (9)	0.0235 (8)	0.0083 (7)	0.0062 (6)	−0.0033 (7)
C31	0.0188 (7)	0.0198 (8)	0.0271 (8)	−0.0049 (6)	0.0037 (6)	−0.0067 (7)
C32	0.0342 (9)	0.0162 (8)	0.0427 (10)	−0.0041 (7)	0.0059 (8)	−0.0002 (8)

### Geometric parameters (Å, °)

**Table:**

Zn1—Zn1^i^	2.8874 (3)	C14—H14A	0.95
Zn1—O1	2.0349 (10)	C15—C16	1.407 (2)
Zn1—O2	2.0251 (10)	C15—H15A	0.95
Zn1—O3	2.0465 (10)	C16—C17	1.414 (2)
Zn1—O4	2.0337 (10)	C17—C18	1.376 (2)
Zn1—N1	2.0484 (12)	C17—H17A	0.95
O1—C1	1.2664 (18)	C18—H18A	0.95
O2—C12	1.2673 (18)	C19—C20	1.504 (4)
O3—C12^i^	1.2599 (18)	C19—H19A	0.99
O4—C1^i^	1.2599 (17)	C19—H19B	0.99
O5—C28	1.2339 (17)	C19A—C20A	1.514 (7)
N1—C23	1.3439 (18)	C19A—H19C	0.99
N1—C27	1.3403 (19)	C19A—H19D	0.99
N2—C28	1.3415 (17)	C20—H20A	0.98
N2—C29	1.4721 (18)	C20—H20B	0.98
N2—C31	1.4678 (19)	C20—H20C	0.98
N3—C5	1.3760 (17)	C20A—H20D	0.98
N3—C8	1.4620 (18)	C20A—H20E	0.98
N3—C10	1.4612 (18)	C20A—H20F	0.98
N4—C16	1.370 (2)	C21—C22	1.508 (3)
N4—C19	1.488 (3)	C21—H21A	0.99
N4—C19A	1.515 (6)	C21—H21B	0.99
N4—C21	1.472 (2)	C21A—C22A	1.514 (8)
N4—C21A	1.527 (8)	C21A—H21C	0.99
C1—O4^i^	1.2599 (17)	C21A—H21D	0.99
C1—C2	1.4896 (19)	C22—H22A	0.98
C2—C3	1.391 (2)	C22—H22B	0.98
C2—C7	1.390 (2)	C22—H22C	0.98
C3—C4	1.3826 (19)	C22A—H22D	0.98
C3—H3A	0.95	C22A—H22E	0.98
C4—C5	1.413 (2)	C22A—H22F	0.98
C4—H4A	0.95	C23—C24	1.382 (2)
C5—C6	1.412 (2)	C23—H23A	0.95
C6—C7	1.3845 (19)	C24—C25	1.393 (2)
C6—H6A	0.95	C24—C28	1.509 (2)
C7—H7A	0.95	C25—C26	1.382 (2)
C8—C9	1.526 (2)	C25—H25A	0.95
C8—H8A	0.99	C26—C27	1.383 (2)
C8—H8B	0.99	C26—H26A	0.95
C9—H9A	0.98	C27—H27A	0.95
C9—H9B	0.98	C29—C30	1.512 (2)
C9—H9C	0.98	C29—H29A	0.99
C10—C11	1.524 (2)	C29—H29B	0.99
C10—H10A	0.99	C30—H30A	0.98
C10—H10B	0.99	C30—H30B	0.98
C11—H11A	0.98	C30—H30C	0.98
C11—H11B	0.98	C31—C32	1.518 (2)
C11—H11C	0.98	C31—H31A	0.99
C12—O3^i^	1.2599 (18)	C31—H31B	0.99
C12—C13	1.486 (2)	C32—H32A	0.98
C13—C14	1.388 (2)	C32—H32B	0.98
C13—C18	1.391 (2)	C32—H32C	0.98
C14—C15	1.377 (2)		
			
O2—Zn1—O4	89.12 (4)	C18—C17—C16	120.93 (15)
O2—Zn1—O1	89.50 (4)	C18—C17—H17A	119.5
O4—Zn1—O1	161.75 (4)	C16—C17—H17A	119.5
O2—Zn1—O3	161.66 (4)	C17—C18—C13	121.68 (14)
O4—Zn1—O3	88.54 (4)	C17—C18—H18A	119.2
O1—Zn1—O3	87.08 (4)	C13—C18—H18A	119.2
O2—Zn1—N1	103.03 (4)	N4—C19—C20	108.2 (3)
O4—Zn1—N1	96.87 (4)	N4—C19—H19A	110.1
O1—Zn1—N1	101.17 (4)	C20—C19—H19A	110.1
O3—Zn1—N1	95.31 (4)	N4—C19—H19B	110.1
O2—Zn1—Zn1^i^	85.53 (3)	C20—C19—H19B	110.1
O4—Zn1—Zn1^i^	80.95 (3)	H19A—C19—H19B	108.4
O1—Zn1—Zn1^i^	80.80 (3)	C19—C20—H20A	109.5
O3—Zn1—Zn1^i^	76.14 (3)	C19—C20—H20B	109.5
N1—Zn1—Zn1^i^	171.18 (3)	H20A—C20—H20B	109.5
C1—O1—Zn1	126.55 (9)	C19—C20—H20C	109.5
C12—O2—Zn1	121.15 (9)	H20A—C20—H20C	109.5
C12^i^—O3—Zn1	132.36 (10)	H20B—C20—H20C	109.5
C1^i^—O4—Zn1	126.57 (10)	C20A—C19A—N4	98.6 (6)
C27—N1—C23	118.75 (12)	C20A—C19A—H19C	112.0
C27—N1—Zn1	120.95 (9)	N4—C19A—H19C	112.0
C23—N1—Zn1	120.09 (10)	C20A—C19A—H19D	112.0
C28—N2—C31	117.63 (12)	N4—C19A—H19D	112.0
C28—N2—C29	124.34 (12)	H19C—C19A—H19D	109.7
C31—N2—C29	117.84 (11)	C19A—C20A—H20D	109.5
C5—N3—C10	120.74 (12)	C19A—C20A—H20E	109.5
C5—N3—C8	120.81 (12)	H20D—C20A—H20E	109.5
C10—N3—C8	117.13 (11)	C19A—C20A—H20F	109.5
C16—N4—C21	121.25 (15)	H20D—C20A—H20F	109.5
C16—N4—C19	121.07 (16)	H20E—C20A—H20F	109.5
C21—N4—C19	117.63 (16)	N4—C21—C22	110.67 (18)
C16—N4—C19A	120.6 (4)	N4—C21—H21A	109.5
C21—N4—C19A	110.3 (4)	C22—C21—H21A	109.5
C16—N4—C21A	116.7 (5)	N4—C21—H21B	109.5
C19—N4—C21A	108.9 (5)	C22—C21—H21B	109.5
C19A—N4—C21A	122.4 (6)	H21A—C21—H21B	108.1
O4^i^—C1—O1	125.11 (13)	C21—C22—H22A	109.5
O4^i^—C1—C2	117.60 (13)	C21—C22—H22B	109.5
O1—C1—C2	117.28 (12)	H22A—C22—H22B	109.5
C7—C2—C3	117.82 (12)	C21—C22—H22C	109.5
C7—C2—C1	121.46 (13)	H22A—C22—H22C	109.5
C3—C2—C1	120.71 (13)	H22B—C22—H22C	109.5
C4—C3—C2	121.84 (13)	C22A—C21A—N4	92.2 (9)
C4—C3—H3A	119.1	C22A—C21A—H21C	113.2
C2—C3—H3A	119.1	N4—C21A—H21C	113.2
C3—C4—C5	120.69 (13)	C22A—C21A—H21D	113.2
C3—C4—H4A	119.7	N4—C21A—H21D	113.2
C5—C4—H4A	119.7	H21C—C21A—H21D	110.6
N3—C5—C6	121.70 (13)	C21A—C22A—H22D	109.5
N3—C5—C4	121.37 (13)	C21A—C22A—H22E	109.5
C6—C5—C4	116.93 (12)	H22D—C22A—H22E	109.5
C7—C6—C5	121.17 (13)	C21A—C22A—H22F	109.5
C7—C6—H6A	119.4	H22D—C22A—H22F	109.5
C5—C6—H6A	119.4	H22E—C22A—H22F	109.5
C6—C7—C2	121.32 (13)	N1—C23—C24	122.65 (13)
C6—C7—H7A	119.3	N1—C23—H23A	118.7
C2—C7—H7A	119.3	C24—C23—H23A	118.7
N3—C8—C9	115.68 (13)	C23—C24—C25	118.02 (13)
N3—C8—H8A	108.4	C23—C24—C28	121.06 (13)
C9—C8—H8A	108.4	C25—C24—C28	120.39 (13)
N3—C8—H8B	108.4	C26—C25—C24	119.61 (13)
C9—C8—H8B	108.4	C26—C25—H25A	120.2
H8A—C8—H8B	107.4	C24—C25—H25A	120.2
C8—C9—H9A	109.5	C25—C26—C27	118.67 (14)
C8—C9—H9B	109.5	C25—C26—H26A	120.7
H9A—C9—H9B	109.5	C27—C26—H26A	120.7
C8—C9—H9C	109.5	N1—C27—C26	122.28 (13)
H9A—C9—H9C	109.5	N1—C27—H27A	118.9
H9B—C9—H9C	109.5	C26—C27—H27A	118.9
N3—C10—C11	113.65 (12)	O5—C28—N2	122.70 (13)
N3—C10—H10A	108.8	O5—C28—C24	118.24 (12)
C11—C10—H10A	108.8	N2—C28—C24	119.05 (12)
N3—C10—H10B	108.8	N2—C29—C30	112.26 (12)
C11—C10—H10B	108.8	N2—C29—H29A	109.2
H10A—C10—H10B	107.7	C30—C29—H29A	109.2
C10—C11—H11A	109.5	N2—C29—H29B	109.2
C10—C11—H11B	109.5	C30—C29—H29B	109.2
H11A—C11—H11B	109.5	H29A—C29—H29B	107.9
C10—C11—H11C	109.5	C29—C30—H30A	109.5
H11A—C11—H11C	109.5	C29—C30—H30B	109.5
H11B—C11—H11C	109.5	H30A—C30—H30B	109.5
O3^i^—C12—O2	124.80 (14)	C29—C30—H30C	109.5
O3^i^—C12—C13	117.40 (13)	H30A—C30—H30C	109.5
O2—C12—C13	117.79 (13)	H30B—C30—H30C	109.5
C14—C13—C18	117.65 (14)	N2—C31—C32	113.34 (13)
C14—C13—C12	120.99 (13)	N2—C31—H31A	108.9
C18—C13—C12	121.36 (13)	C32—C31—H31A	108.9
C15—C14—C13	121.76 (15)	N2—C31—H31B	108.9
C15—C14—H14A	119.1	C32—C31—H31B	108.9
C13—C14—H14A	119.1	H31A—C31—H31B	107.7
C14—C15—C16	121.05 (15)	C31—C32—H32A	109.5
C14—C15—H15A	119.5	C31—C32—H32B	109.5
C16—C15—H15A	119.5	H32A—C32—H32B	109.5
N4—C16—C15	121.43 (15)	C31—C32—H32C	109.5
N4—C16—C17	121.65 (15)	H32A—C32—H32C	109.5
C15—C16—C17	116.92 (14)	H32B—C32—H32C	109.5
			
O2—Zn1—O1—C1	86.39 (12)	O2—C12—C13—C18	177.38 (13)
O4—Zn1—O1—C1	0.7 (2)	C18—C13—C14—C15	1.0 (2)
O3—Zn1—O1—C1	−75.59 (12)	C12—C13—C14—C15	−177.97 (15)
N1—Zn1—O1—C1	−170.43 (12)	C13—C14—C15—C16	−0.5 (3)
Zn1^i^—Zn1—O1—C1	0.84 (11)	C21—N4—C16—C15	−9.2 (3)
O4—Zn1—O2—C12	80.83 (11)	C19—N4—C16—C15	168.0 (2)
O1—Zn1—O2—C12	−80.97 (11)	C19A—N4—C16—C15	−155.2 (4)
O3—Zn1—O2—C12	−1.8 (2)	C21A—N4—C16—C15	31.6 (6)
N1—Zn1—O2—C12	177.68 (10)	C21—N4—C16—C17	169.95 (19)
Zn1^i^—Zn1—O2—C12	−0.16 (10)	C19—N4—C16—C17	−12.8 (3)
O2—Zn1—O3—C12^i^	3.0 (2)	C19A—N4—C16—C17	24.0 (5)
O4—Zn1—O3—C12^i^	−79.71 (13)	C21A—N4—C16—C17	−149.3 (5)
O1—Zn1—O3—C12^i^	82.56 (13)	C14—C15—C16—N4	178.53 (19)
N1—Zn1—O3—C12^i^	−176.48 (13)	C14—C15—C16—C17	−0.7 (3)
Zn1^i^—Zn1—O3—C12^i^	1.32 (12)	N4—C16—C17—C18	−177.90 (19)
O2—Zn1—O4—C1^i^	−84.71 (12)	C15—C16—C17—C18	1.3 (3)
O1—Zn1—O4—C1^i^	1.0 (2)	C16—C17—C18—C13	−0.8 (3)
O3—Zn1—O4—C1^i^	77.11 (12)	C14—C13—C18—C17	−0.4 (2)
N1—Zn1—O4—C1^i^	172.28 (12)	C12—C13—C18—C17	178.60 (15)
Zn1^i^—Zn1—O4—C1^i^	0.91 (12)	C16—N4—C19—C20	92.3 (3)
O2—Zn1—N1—C27	−56.86 (11)	C21—N4—C19—C20	−90.3 (2)
O4—Zn1—N1—C27	33.82 (11)	C19A—N4—C19—C20	−6.4 (7)
O1—Zn1—N1—C27	−148.95 (10)	C21A—N4—C19—C20	−128.3 (5)
O3—Zn1—N1—C27	122.98 (10)	C16—N4—C19A—C20A	−99.3 (6)
O2—Zn1—N1—C23	128.42 (10)	C21—N4—C19A—C20A	111.4 (5)
O4—Zn1—N1—C23	−140.90 (10)	C19—N4—C19A—C20A	1.3 (4)
O1—Zn1—N1—C23	36.33 (11)	C21A—N4—C19A—C20A	73.6 (8)
O3—Zn1—N1—C23	−51.73 (10)	C16—N4—C21—C22	88.0 (2)
Zn1—O1—C1—O4^i^	−1.8 (2)	C19—N4—C21—C22	−89.4 (3)
Zn1—O1—C1—C2	177.44 (9)	C19A—N4—C21—C22	−123.0 (4)
O4^i^—C1—C2—C7	−2.5 (2)	C21A—N4—C21—C22	−5.3 (8)
O1—C1—C2—C7	178.20 (13)	C16—N4—C21A—C22A	−98.4 (8)
O4^i^—C1—C2—C3	176.53 (13)	C21—N4—C21A—C22A	8.8 (6)
O1—C1—C2—C3	−2.8 (2)	C19—N4—C21A—C22A	120.2 (8)
C7—C2—C3—C4	4.3 (2)	C19A—N4—C21A—C22A	88.5 (9)
C1—C2—C3—C4	−174.77 (13)	C27—N1—C23—C24	−0.2 (2)
C2—C3—C4—C5	−1.2 (2)	Zn1—N1—C23—C24	174.59 (10)
C10—N3—C5—C6	163.01 (13)	N1—C23—C24—C25	0.6 (2)
C8—N3—C5—C6	−3.5 (2)	N1—C23—C24—C28	−171.13 (12)
C10—N3—C5—C4	−17.2 (2)	C23—C24—C25—C26	0.1 (2)
C8—N3—C5—C4	176.24 (13)	C28—C24—C25—C26	171.85 (13)
C3—C4—C5—N3	177.03 (13)	C24—C25—C26—C27	−1.0 (2)
C3—C4—C5—C6	−3.2 (2)	C23—N1—C27—C26	−0.8 (2)
N3—C5—C6—C7	−175.71 (14)	Zn1—N1—C27—C26	−175.60 (10)
C4—C5—C6—C7	4.5 (2)	C25—C26—C27—N1	1.5 (2)
C5—C6—C7—C2	−1.5 (2)	C31—N2—C28—O5	−2.0 (2)
C3—C2—C7—C6	−2.9 (2)	C29—N2—C28—O5	172.87 (13)
C1—C2—C7—C6	176.11 (14)	C31—N2—C28—C24	176.98 (12)
C5—N3—C8—C9	82.09 (17)	C29—N2—C28—C24	−8.2 (2)
C10—N3—C8—C9	−84.90 (15)	C23—C24—C28—O5	106.09 (16)
C5—N3—C10—C11	−74.11 (17)	C25—C24—C28—O5	−65.45 (18)
C8—N3—C10—C11	92.88 (15)	C23—C24—C28—N2	−72.92 (18)
Zn1—O2—C12—O3^i^	−0.69 (19)	C25—C24—C28—N2	115.54 (15)
Zn1—O2—C12—C13	178.81 (9)	C28—N2—C29—C30	114.21 (15)
O3^i^—C12—C13—C14	175.86 (14)	C31—N2—C29—C30	−70.95 (16)
O2—C12—C13—C14	−3.7 (2)	C28—N2—C31—C32	−76.65 (17)
O3^i^—C12—C13—C18	−3.1 (2)	C29—N2—C31—C32	108.16 (15)

Symmetry codes: (i) −*x*+1, −*y*, −*z*.

### Hydrogen-bond geometry (Å, °)

**Table:**

*D*—H···*A*	*D*—H	H···*A*	*D*···*A*	*D*—H···*A*
C10—H10A···O5^ii^	0.99	2.49	3.390 (2)	151
C21—H21B···Cg1^iii^	0.99	2.94	3.879 (3)	163
C29—H29A···Cg1^iv^	0.99	2.87	3.637 (2)	137

Symmetry codes: (ii) *x*+1/2, −*y*+1/2, *z*−1/2; (iii) *x*, *y*+1, *z*; (iv) −*x*, −*y*, −*z*.
